# Determination of Delafloxacin in Pharmaceutical Formulations Using a Green RP-HPTLC and NP-HPTLC Methods: A Comparative Study

**DOI:** 10.3390/antibiotics9060359

**Published:** 2020-06-25

**Authors:** Prawez Alam, Essam Ezzeldin, Muzaffar Iqbal, Gamal A.E. Mostafa, Md. Khalid Anwer, Mohammed H. Alqarni, Ahmed I. Foudah, Faiyaz Shakeel

**Affiliations:** 1 Department of Pharmacognosy, College of Pharmacy, Prince Sattam Bin Abdulaziz University, Al-Kharj 11942, Saudi Arabia; p.alam@psau.edu.sa (P.A.); m.alqarni@psau.edu.sa (M.H.A.); a.foudah@psau.edu.sa (A.I.F.); 2 Department of Pharmaceutical Chemistry, College of Pharmacy, King Saud University, Riyadh 11451, Saudi Arabia; ezzeldin24@hotmail.com (E.E.); muziqbal@gmail.com (M.I.); gamal_most@yahoo.com (G.A.E.M.); 3 Drug Bioavailability Unit, Central Laboratory, College of Pharmacy, King Saud University, P.O. Box 2457, Riyadh 11451, Saudi Arabia; 4 Micro-Analytical Laboratory, Applied Organic Chemistry Department, National Research Center, Dokki, Cairo 12622, Egypt; 5 Department of Pharmaceutics, College of Pharmacy, Prince Sattam Bin Abdulaziz University, Al-Kharj 11942, Saudi Arabia; mkanwer2002@yahoo.co.in; 6 Department of Pharmaceutics, College of Pharmacy, King Saud University, Riyadh 11451, Saudi Arabia

**Keywords:** delafloxacin, green RP-HPTLC, NP-HPTLC, ICH guidelines, solid lipid nanoparticles

## Abstract

In this work; delafloxacin (DLFX) was determined using a validated green RP-HPTLC and NP-HPTLC methods in commercial tablets and in-house developed solid lipid nanoparticles (SLNs). RP-HPTLC determination of DLFX was performed using “RP-18 silica gel 60 F254S HPTLC plates”. However; NP-HPTLC estimation of DLFX was performed using “silica gel 60 F254S HPTLC plates”. For a green RP-HPTLC method; the ternary combination of ethanol:water:ammonia solution (5:4:2 *v*/*v*/*v*) was used as green mobile phase. However; for NP-HPTLC method; the ternary mixture of ethyl acetate: methanol: ammonia solution (5:4:2 *v*/*v*/*v*) was used as normal mobile phase. The analysis of DLFX was conducted in absorbance/reflectance mode of densitometry at *λ*_max_ = 295 nm for both methods. RP-HPTLC method was found more accurate, precise, robust and sensitive for the analysis of DLFX compared with the NP-HPTLC method. The % assay of DLFX in commercial tablets and in-house developed SLNs was determined as 98.2 and 101.0%, respectively, using the green RP-HPTLC technique, however; the % assay of DLFX in commercial tablets and in-house developed SLNs was found to be 94.4 and 95.0%, respectively, using the NP-HPTLC method. Overall, the green RP-HPTLC method was found superior over the NP-HPTLC. Therefore, the proposed green RP-HPTLC method can be successfully applied for analysis of DLFX in commercial tablets, SLNs and other formulations containing DLFX.

## 1. Introduction

Delafloxacin (DLFX) is a novel fluoroquinolone antibiotic which was approved by the US FDA in 2017 [1,2] for the treatment of “acute bacterial skin and skin structure infections (ABSSSIs)” [3,4]. It has also been found effective for the treatment of community-acquired respiratory tract infections (CARTIs) [4,5]. It is under clinical trial for CARTIs treatment [5]. It has been reported active against major microbes responsible for ABSSSIs and CARTIs [6,7]. It is rapidly absorbed following oral administration with peak plasma levels obtained within 1−2.5 h [8,9]. The absolute bioavailability of DLFX was found to be very low (58.8%) which was probably due to its poor solubility in aqueous media such as water [10]. A literature survey revealed a single derivative spectroscopic method for the determination of DLFX in bulk materials and laboratory prepared mixtures [11]. A single “ultra-high performance liquid chromatography tandem mass spectrometry/mass spectrometry (UHPLC-MS/MS)” method has also been reported for the estimation of DLFX in rat plasma and rabbit aqueous humour but not in pharmaceutical dosage forms [10]. However, the cost and maintenance of more robust and reliable apparatus such as “UHPLC-MS/MS” apparatus is very high compared with “high-performance thin layer chromatography (HPTLC)”. Moreover, highly-skilled trained personnel are required to operate “UHPLC-MS/MS” apparatus compared with HPTLC [10,12]. Recently, a HPTLC method has been reported for the estimation of DLFX in human plasma and its pharmacokinetic assessment in real plasma samples of rats [12]. Although, the reported HPTLC method was validated for the estimation of DLFX in biological samples, it was not applied to real pharmaceutical formulation samples [12]. Some clinical reports are also available for the assessment of pharmacokinetic profile of DLFX in healthy human subjects and diseased patients but these clinical reports have not disclosed any information about method development and validation [13,14,15]. To the best of our knowledge, not a single HPTLC method has been applied for the determination of DLFX in its pure form and pharmaceutical formulations. Pharmaceutical analysis laboratories and pharmaceutical industries are using huge number of toxic solvents such as acetonitrile, methanol and chloroform etc. either alone or in combination for pharmaceutical and bioanalytical procedures which causes the significant problems to the environment due to their environmental toxicity [16]. In addition, the environmental effects of analytical and bioanalytical procedures have been either neglected or little attention has been paid towards such analysis [16,17]. In recent years, mentions in the literature of analytical and bioanalytical procedures related with “green analytical chemistry or environmentally-benign analytical methods’ have increased significantly [18,19,20,21,22]. In spite of their various beneficial characteristics, including “non-toxicity, non-volatility, non-inflammability, non-aggressiveness, high biodegradability and cost effectiveness”, the full potential of green solvents had not been utilized fully as the mobile phase for HPTLC estimation of drugs/pharmaceuticals [23,24,25].

Green HPTLC techniques offer several advantages compared with conventional HPTLC techniques in pharmaceutical/biomedical analysis [26,27,28,29,30]. Therefore, in this research; DLFX was estimated using a green reversed phase HPTLC (RP-HPTLC) method in commercial tablets and in-house developed solid lipid nanoparticles (SLNs). For comparison purposes, DLFX was also quantified in commercial tablets and in-house developed SLNs using a normal phase HPTLC (NP-HPTLC) method. In general, green analytical methods suffer sensitivity problems in pharmaceutical analysis compared with routine analytical techniques [16,17]. In order to compare various validation parameters of the proposed green RP-HPTLC technique, the NP-HPTLC method was also used as a standard method for the estimation of DLFX. Both green RP-HPTLC and NP-HPTLC methods were validated in terms of linearity, accuracy, precision, robustness and sensitivity according to the “International Conference on Harmonization (ICH)” Q2 (R1) guidelines [31].

## 2. Materials and Methods

### 2.1. Materials Used

DLFX was procured from Beijing Mesochem Technology Pvt. Ltd. (Beijing, China). Chitosan (CS) and stearic acid (SA) were acquired from Sigma Aldrich (St. Louis, MO, USA). Chromatography grades solvents (i.e., methanol, ethanol and ethyl acetate), ammonia solution (30% *v/v*) and glacial acetic acid were obtained from E. Merck (Darmstadt, Germany). Pluronic F-127 was obtained from BASF (Ludwigshafen, Germany). Deionized water was collected from a Milli-Q unit. Commercial tablets of DLFX were obtained from a pharmacy in Riyadh, Saudi Arabia. All other solvents and reagents were of analytical reagent grades.

### 2.2. Preparation of Stock Slutions and DLFX Calibration Curve 

Standard stock solution (SSS) of DLFX was obtained by dissolving an accurately weighed 10 mg sample of DLFX in 10 mL of methanol. Around 1 mL of SSS of DLFX was further diluted with mobile phase in order to obtain the final SSS of 100 μg/mL. Different volumes of SSS (100 μg/mL) were taken and serial dilutions were made in order to obtain concentrations in the range of 25−1000 and 50−600 ng/band of DLFX for RP-HPTLC and NP-HPTLC, respectively. These concentrations of DLFX were spotted on TLC plates and the peak area of DLFX for each concentration was recorded for both methods. The calibration curve (CC) of DLFX was constructed between the concentration and measured area for both methods. The CC for a green RP-HPTLC method was obtained in the range of 25−1000 ng/band. However, the CC for a NP-HPTLC method was obtained in the range of 50−600 ng/band.

### 2.3. Sample Preparation for the Analysis of DLFX in Commercial Tablets 

Ten commercial tablets (each tablet containing 450 mg of DLFX) were taken, weighed and the average weight was determined. The tablets were crushed and a fine powder was obtained. A weighed sample of the fine powder containing 50 mg of DLFX was dissolved in methanol and diluted with mobile phase in order to obtain 100 mL stock solution (SS) for both methods. This SS was filtered to eliminate any insoluble substance and sonicated for about 10 min. Around 1.0 mL of this SS of commercial tablets was diluted further with 10 mL of mobile phase for both methods. The diluted SS was taken for the analysis of DLFX content in commercial tablets using RP-HPTLC and NP-HPTLC methods.

### 2.4. Preparation of and Characterization DLFX-Loaded SLNs

DLFX-loaded SA-CS-SLNs were prepared by applying a “single emulsion solvent evaporation method” [32]. In the preparation of SLNs, SA, Pluronic F-127 and CS were used as lipid, surfactant and polymer, respectively. The organic phase was obtained by dissolving 400 mg of SA in 2 mL of ethyl acetate. An accurately weighed 10 mg sample of DLFX was dissolved in 2 mL of organic phase i.e., SA solution in ethyl acetate. The aqueous phase was prepared by dissolving 10 mg of CS in 1% *w*/*v* aqueous acetic acid solution (1 mg/mL) containing 50 mg of Pluronic F-127. The organic and aqueous phases were emulsified for about 3 min at 60% voltage efficiency with the help of a probe sonicator (Ultrasonic Processor, gx-130, Berlin, Germany) at 25 °C. The volatile organic solvent was evaporated by magnetic stirrer at 40 °C for overnight. After complete evaporation of ethyl acetate, DLFX-loaded SA-CS-SLNs were separated from the bulk aqueous phase using high speed centrifugation (Hermle Labortechnik, Wehingen, Germany) at 10,500× *g* for about 30 min followed by three times washing with cold distilled water and finally freeze dried using a Freeze Dryer (Millirock Technology, Kingston, NY, USA). DLFX-loaded SA-CS-SLNs were characterized in terms of particle size, polydispersity index, zeta potential and entrapment efficiency [32].

### 2.5. Sample Preparation for Determination of DLFX in In-House Developed SLNs 

Approximately 250 mg of in-house developed SLNs (containing 50 mg of DLFX) were dissolved in methanol and diluted with mobile phase in order to obtain 50 mL SS for both methods. The SS of DLFX was filtered in order to eliminate insoluble materials and sonicated for about 10 min. About 1.0 mL of above SS was further diluted with 10 mL of mobile phase for both methods. The diluted SS was subjected for the estimation of DLFX in in-house developed SLNs using green RP-HPTLC and NP-HPTLC methods.

### 2.6. Instrumentation and Analytical Conditions 

For the determination of DLFX by green RP-HPTLC and NP-HPTLC methods, the following instrumentation and analytical conditions were applied:

HPTLC instrument: CAMAG TLC system (Muttenz, Basel-landschaft, Switzerland)
Software: WinCAT (version 1.4.3.6336)
Syringe for sample application: CAMAG microliter Syringe (Hamilton, Bonaduz, Switzerland)
TLC plates: 10 × 20 cm glass backed plates pre-coated with RP-18 silica gel 60 F254S plates (E-Merck, Darmstadt, Germany) for RP-HPTLC method and 10 × 20 cm glass backed plates pre-coated with silica gel 60 F254S plates (E. Merck) for NP-HPTLC method
Sample applicator: CAMAG Linomat-V
Gas for sample application: Nitrogen
Development chamber: CAMAG automatic developing chamber 2 (ADC2)
TLC scanner: CAMAG TLC scanner-III
Stationary phase: 10 × 20 cm glass backed plates pre-coated with RP-18 silica gel 60 F254S plates (E. Merck) for the RP-HPTLC method and 10 × 20 cm glass backed plates pre-coated with silica gel 60 F254S plates (E. Merck) for the NP-HPTLC method
Mobile phase for a green RP-HPTLC method: ethanol:water:ammonia solution (5:4:2 *v/v/v*)
Mobile phase for NP-HPTLC method: Ethyl acetate: methanol: ammonia solution (5:4:2 *v/v/v*)
Saturation time of mobile phase: 30 min at 22 °C
Development distance on plate: 80 mm
Development mode: Linear ascending mode
Sample application rate: 150 nL/s
Densitometry of scanning mode: Absorbance/reflectance.
Scanning wavelength of APM: 295 nm

### 2.7. Analytical Method Validation

Both green RP-HPTLC and NP-HPTLC methods for the determination of DLFX were validated in terms of “linearity, precision, accuracy, robustness, sensitivity and specificity” as per ICH Q2 (R1) guidelines [31]. The linearity of DLFX was determined by plotting the concentration of DLFX against the measured HPTLC area of DLFX. Linearity was found out in the concentration range of 25−1000 ng/band and 50−600 ng/band for the RP-HPTLC and NP-HPTLC, respectively. The method accuracy was found out as the percent of recovery (% recovery) at four different concentrations of DLFX. As the linearity range was different for NP-HPTLC and RP-HPTLC methods, the studied concentrations for accuracy measurement were different for both methods. For the RP-HPTLC method, the % recovery of DLFX was estimated at four different concentrations (75, 300, 400 and 500 ng/band). However, for the NP-HPTLC method, the % recovery was estimated at four different concentrations of 150, 300, 400 and 500 ng/band. The studied concentrations for accuracy measurements were selected as per ICH guidelines [31]. 

Method precision was measured as repeatability and intermediate precision. Repeatability, i.e., intra-day precision, was determined by the analysis of samples on the same day at 75, 300 and 400 ng/band concentrations (*n* = 6) of DLFX for the RP-HPTLC method. The repeatability of the NP-HPTLC method was determined at 150, 300 and 400 ng/band concentrations (*n* = 6) of DLFX. Intermediate/inter-day precision was obtained by the analysis of samples on three consecutive days at 75, 300 and 400 ng/band concentrations (*n* = 6) of DLFX for the RP-HPTLC method. The intermediate precision of the NP-HPTLC method was also determined on three consecutive days at 150, 300 and 400 ng/band concentrations (*n* = 6) of DLFX. According to ICH guidelines for precision measurement, at least three different concentrations within the linearity range should be analyzed [31]. Therefore, the proposed concentrations were selected for precision evaluation.

Method robustness was found out by making a small deliberate variation in the composition of the mobile phase during DLFX analysis. For the green RP-HPTLC method, the original mobile phase composition of ethanol:water:ammonia solution (5:4:2, *v/v/v*) was changed to ethanol:water:ammonia solution (5.2:3.8:2, *v/v/v*) and ethanol:water:ammonia solution (4.8:4.2:2, *v/v/v*) for the positive and negative level, respectively. For the NP-HPTLC method, the original mobile phase composition of ethyl acetate: methanol: ammonia solution (5:4:2, *v/v/v*) was changed to ethyl acetate: methanol: ammonia solution (5.2:3.8:2, *v/v/v*) and ethyl acetate: methanol: ammonia solution (4.8:4.2:2, *v/v/v*) for the positive and negative level, respectively. 

Method sensitivity was found out in terms of “limit of detection (LOD) and limit of quantification (LOQ)” using “standard deviation (SD)” technique. The “LOD and LOQ” values of DLFX for both methods were calculated using Equations (1) and (2), respectively:

(1) $$\mathrm{LOD}=3.3\times \frac{\mathrm{SD}}{S}$$

(2) $$\mathrm{LOQ}=10\times \frac{\mathrm{SD}}{S}$$

where, S = slope of the CC of DLFX. 

The method specificity for both methods was determined by comparing the R_f_ values and UV absorption spectra of DLFX in SLNs samples with that of the standard DLFX.

For the analysis of DLFX in commercial tablets and SLNs the samples of commercial tablets and in-house developed SLNs were applied on TLC plates and HPTLC-densitograms were recorded using the same experimental procedures as described for the determination of standard DLFX. The HPTLC area of DLFX in tablet dosage forms and in-house developed SLNs was determined. The amount of DLFX in both formulations was calculated using the CC of DLFX for both methods.

## 3. Results and Discussion

### 3.1. Preparation and Characterization of DLFX-Loaded SLNs

DLFX-loaded SA-CS-SLNs were obtained using a “single emulsion solvent evaporation method” [32]. The composition of SA-CS-SLNs and their characterization parameters are summarized in Table 1. The particle size and polydispersity index of SA-CS-SLNs were found as 368.0 ± 5.2 nm and 0.2 ± 0.0, respectively. The zeta potential of SA-CS-SLNs was found to be 19.2 ± 1.4 mV. The entrapment efficiency of SA-CS-SLNs was obtained as 80.4 ± 3.1%. These results indicated that the SA-CS-SLNs of DLFX was well prepared in the laboratory. 

### 3.2. Method Development

A literature survey indicated not a single RP-HPTLC or NP-HPTLC method for the determination of DLFX in SLNs. Therefore, the present work was aimed to develop a green RP-HPTLC method for the determination of DLFX in commercial tablets and in-house developed SLNs in comparison with a NP-HPTLC method. In the green RP-HPTLC method, the mobile phase was obtained by the simple mixture of ethanol, water and ammonia solution. However, in the case of the NP-HPTLC method, the mobile phase was prepared by mixing ethyl acetate, methanol and ammonia solution. The use of the RP-HPTLC method presents several advantages over NP-HPTLC which include avoidance of the non-polar fractions from the sample in the TLC plates, avoidance of the interference due to the presence of impurities, formation of compact spot and detection clarity [16,33]. The green RP-HPTLC method for the determination of DLFX will also decrease the chances of toxicity to the environment compared with the NP-HPTLC method [26,27]. 

**Figure 1:**
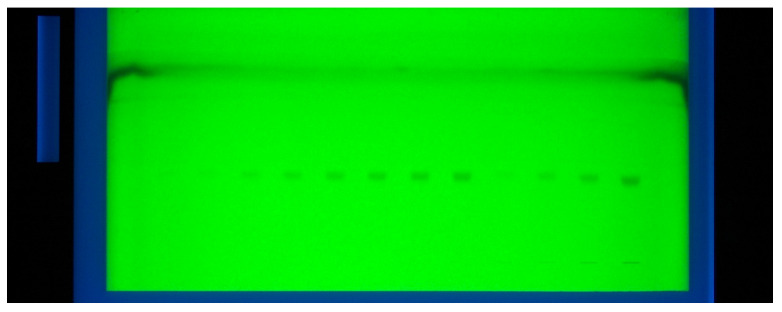
Pictorial diagram for the developed TLC plate for DLFX analysis.

In this study, various compositions of ethanol:water:ammonia solution such as 6:4:1 (*v/v/v*), 5:5:1 (*v/v/v*), 4:5:2 (*v/v/v*), 4:4:3 (*v/v/v*) and 5:4:2 (*v/v/v*) were studied as the mobile phase for the development of a suitable band for RP-HPTLC-densitometric analysis of DLFX. The green mobile phase was developed using chamber saturation conditions (Figure 1). 

**Figure 2:**
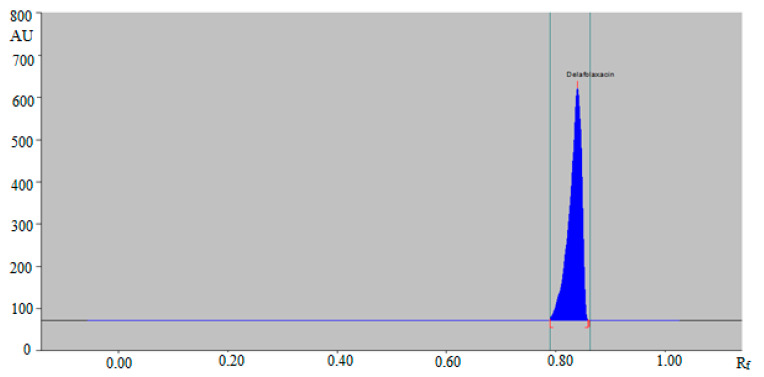
HPTLC chromatogram of standard DLFX by green RP-HPTLC method.

The combination of ethanol:water:ammonia solutions such as 6:4:1 (*v/v/v*) and 5:5:1 (*v/v/v*) resulted in the presentation of poor densitometric peak with a poor symmetry of DLFX. However, the combinations of ethanol:water:ammonia solution such as 4:5:2 (*v/v/v*) and 4:4:3 (*v/v/v*) presented good densitometric peaks but the peak symmetry of DLFX was poor. Out of the various ethanol, water and ammonia solution compositions studied, the ternary mixture of ethanol:water:ammonia solution 5:4:2 (*v/v/v*) gave a well-resolved and compact peak of DLFX at R_f_ = 0.84 ± 0.01 (Figure 2).

**Figure 3:**
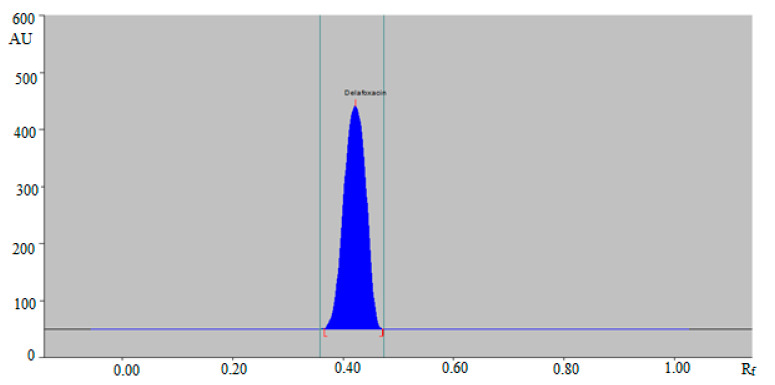
HPTLC chromatogram of standard DLFX by NP-HPTLC method.

Hence, the ternary mixture of ethanol:water:ammonia solution 5:4:2 (*v*/*v/v*) was chosen as the mobile phase for the determination of DLFX in commercial tablets and in-house developed SLNs using the green RP-HPTLC method. Similarly, various compositions of ethyl acetate: methanol: ammonia solution such as 6:4:1 (*v/v/v*), 5:5:1 (*v/v/v*), 4:5:2 (*v/v/v*), 4:4:3 (*v/v/v*) and 5:4:2 (*v/v/v*) were studied as the mobile phase for the development of a suitable band for NP-HPTLC-densitometric analysis of DLFX. The combinations of ethyl acetate: methanol: ammonia solution such as 6:4:1 (*v/v/v*), 5:5:1 (*v/v/v*), 4:5:2 (*v/v/v*) and 4:4:3 (*v/v/v*) gave the same results as recorded for the green RP-HPTLC method. The NP-HPTLC mobile phase was also developed using chamber saturation conditions. Out of various compositions of ethyl acetate, methanol and ammonia solution studied, the ternary mixture of ethyl acetate: methanol: ammonia solution 5:4:2 (*v/v/v*) gave a well-resolved and compact peak of DLFX at R_f_ = 0.44 ± 0.01 (Figure 3). 

Hence, the ternary mixture of ethyl acetate: methanol: ammonia solution 5:4:2 (*v/v/v*) was chosen as the mobile phase for the determination of DLFX in commercial tablets and in-house developed SLNs using the NP-HPTLC method. The bands spectra for both methods were recorded densitometrically and maximum HPTLC response under reflectance/absorbance mode was observed at λ_max_ = 295 nm for both methods. Therefore, all analyses of DLFX were carried out at 295 nm for both methods. 

### 3.3. Method Validation

The results for linear regression analysis of CC of DLFX for both methods are summarized in Table 2. 

The CC of DLFX was found linear in concentration range of 25−1000 ng/band for the green RP-HPTLC method. However, the CC of DLFX was found linear in concentration range of 50−600 ng/band for the NP-HPTLC method. The data for both methods showed good linear relationship between the concentration and HPTLC area (Table 2). The determination coefficient (R^2^) values for DLFX was regressed as 0.9996 and 0.9995 for the green RP-HPTLC and NP-HPTLC methods, respectively.

The R^2^ values for both methods were highly significant (*p* < 0.05). The linear regression equation for the green RP-HPTLC method was computed as Y = 33.62x − 184.11, in which Y is the measured peak area and x represents the concentration of DLFX. The linear regression equation for the NP-HPTLC method was computed as Y = 23.90x + 201.55. Overall, the linear regression analysis suggested good linearity of both the methods. Method accuracy for both methods was computed as % recovery. The results of accuracy evaluation for both methods are presented in Table 3.

The % recovery of DLFX for the green RP-HPTLC method was computed as 98.4−101.0%. The % RSD in the accuracy of DLFX for the green RP-HPTLC method was computed as 1.29−1.89%. The % recovery of DLFX for the NP-HPTLC method was computed as 95.9−96.7%. The % RSD in the accuracy of DLFX for the NP-HPTLC method was computed as 1.73−2.65%. The % recovery of DLFX using various derivative spectrometry methods has been reported as 97.5−100.7% in literature [11]. The recorded % recovery of DLFX (98.4−101.0%) using the green RP-HPTLC method was better than the reported derivative spectrometry method. However, the recorded % recovery of DLFX (95.9−96.7%) using the NP-HPTLC method was much lower than reported derivative spectrometry method. The computed values of accuracy within the limit of 100 ± 2% and % RSD ± 2% suggested that the green RP-HPTLC method was more accurate for the determination of DLFX compared with the NP-HPTLC method.

Method precision for both methods was computed as % RSD. The results of precision for both methods are listed in Table 4. The % RSD values of the green RP-HPTLC method for the repeatability and intermediate precision were computed as 1.14−1.60% and 1.26−1.70%, respectively. The % RSD values of the NP-HPTLC method for the repeatability and intermediate precision were computed as 1.72−2.45% and 1.84−2.61%, respectively. The % RSD values using various derivative spectrometry methods for the repeatability and intermediate precision have been reported as 0.45−1.92% and 0.37−1.54%, respectively in literature [11]. The recorded % RSD values using the green RP-HPTLC method for the repeatability and intermediate precision were similar to reported derivative spectrometry method. However, the recorded % RSD values using the NP-HPTLC method for the repeatability and intermediate precision were much higher compared to reported derivative spectrometry method. The computed values of % RSD within the limit of ±2% indicated that the green RP-HPTLC method was more precise for the determination of DLFX compared with the NP-HPTLC method.

Results of method robustness for both methods are listed in Table 5. The % RSD values after introducing small deliberate changes in the composition of mobile phase were recorded as 1.08−1.21% for the green RP-HPTLC method. The R_f_ value for DLFX after this change was recorded in the range of 0.83−0.85 for the green RP-HPTLC method. However, the % RSD values were recorded as 1.66−1.87% for the NP-HPTLC method. The R_f_ value for DLFX after this change was recorded in the range of 0.43−0.45 for the NP-HPTLC method. The small variations in R_f_ values of DLFX and lower % RSD values indicated that both methods were robust for the determination of DLFX. However, the green RP-HPTLC method was more robust than the NP-HPTLC method. 

The method sensitivity for both methods was computed as “LOD and LOQ”. The computed values of “LOD and LOD” for both methods are presented in Table 2. The “LOD and LOQ” values of the green RP-HPTLC method were computed as 8.54 ± 0.22 and 25.62 ± 0.66 ng/band, respectively for DLFX. However, the “LOD and LOQ” values of the NP-HPTLC method were computed as 17.31 ± 0.51 and 51.93 ± 1.53 ng/band, respectively for DLFX. The computed values of “LOD and LOQ” suggested that both methods were sensitive enough for the determination of DLFX. However, the green RP-HPTLC method was more sensitive than the NP-HPTLC method. 

**Figure 4:**
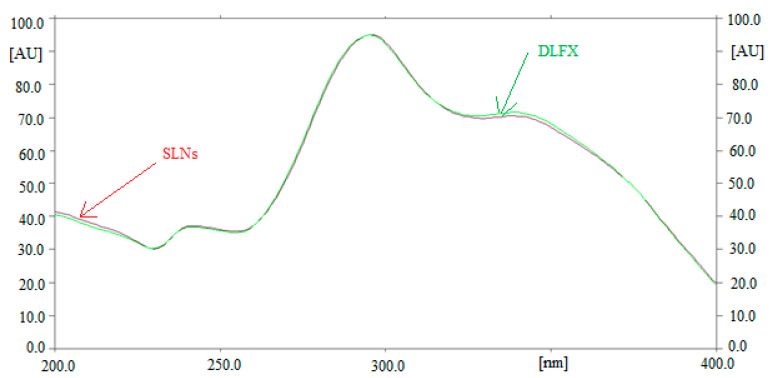
Overlaid UV absorption spectra of standard DLFX and in-house developed SLNs.

Method specificity and the peak purity of DLFX for both methods were obtained by comparing the overlaid spectra of DLFX in SLN samples and standard DLFX. The overlaid spectra of standard DLFX and DLFX in in-house developed SLNs are presented in Figure 4. The maximum densitometric response of DLFX in standard and SLNs was found at *λ_max_* = 295 nm for both methods. The similar HPTLC spectra, *R_f_* values and *λ_max_* of DLFX in standard and SLNs suggested the specificity and peak purity of both methods.

### 3.4. Determination of DLFX in Commercial Tablets and SLNs 

**Figure 5:**
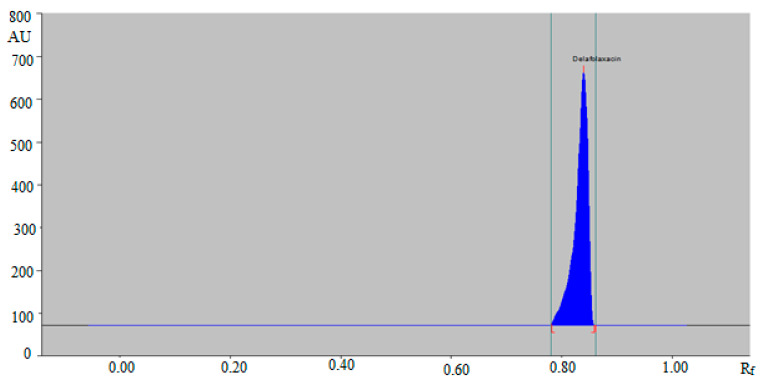
HPTLC chromatogram of DLFX in in-house developed SLNs by the green RP-HPTLC method.

A literature survey indicated not a single green RP-HPTLC method for the determination of DLFX in pharmaceutical products and biological samples. Hence, a green RP-HPTLC method could be an alternative approach for the determination of DLFX in pharmaceutical products compared with a NP-HPTLC method. The HPTLC peak of DLFX from commercial tablet and SLNs was identified by comparing their single TLC spot at *R_f_* = 0.84 ± 0.01 with that of standard DLFX for the green RP-HPTLC method. The representative HPTLC chromatogram of DLFX in in-house developed SLNs for the green RP-HPTLC method is presented in Figure 5, which is found to be similar to that of standard DLFX. 

**Figure 6:**
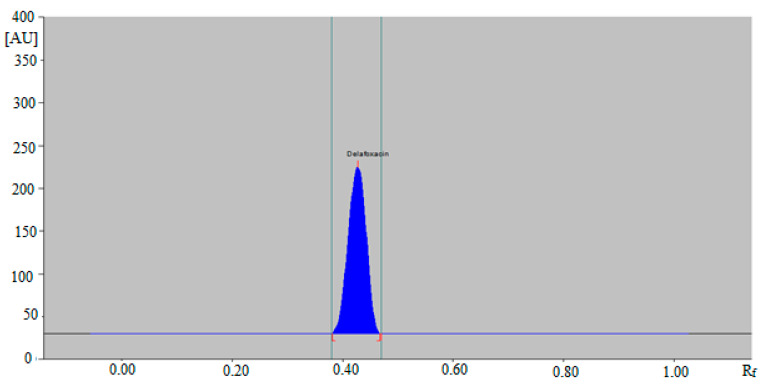
HPTLC chromatogram of DLFX in in-house developed SLNs by NP-HPTLC method.

The HPTLC peak of DLFX from commercial tablets and SLNs was also identified by comparing their single TLC spots at R_f_ = 0.44 ± 0.01 with that of standard DLFX for the NP-HPTLC method. The representative HPTLC chromatogram of DLFX in in-house developed SLNs for the NP-HPTLC method is presented in Figure 6, which is also similar with that of standard DLFX. The amount of DLFX in commercial tablets and in-house developed SLNs was computed by the CC of DLFX. The results of the determination of DLFX in commercial tablets SLNs for both methods are listed in Table 6. 

The amount of DLFX in commercial tablets was computed as 49.1 ± 1.0 mg out of 50 mg of theoretical DLFX using the green RP-HPTLC method. However, the amount of DLFX in in-house developed SLNs was computed as 50.5 ± 1.1 mg compared with 50 mg of theoretical DLFX using the green RP-HPTLC method. The amount of DLFX in commercial tablets was found as 47.2 ± 1.2 mg out of 50 mg of theoretical DLFX using the NP-HPTLC method. However, the amount of DLFX in in-house developed SLNs was found as 47.5 ± 1.3 mg out of 50 mg of theoretical DLFX using the NP-HPTLC method. The % assay of DLFX in commercial tablets and in-house developed SLNs was obtained as 98.2 and 101.0%, respectively using the green RP-HPTLC method. However, the % assay of DLFX in commercial tablets and in-house developed SLNs was obtained as 94.4 and 95.0%, respectively using the NP-HPTLC method. The % assay of DLFX within the range of ±2% by a green RP-HPTLC method suggested that the green RP-HPTLC method can be successfully applied for the pharmaceutical analysis of DLFX in commercial products containing DLFX as an active drug compared with the NP-HPTLC method. Based on accuracy, precision, robustness, sensitivity and pharmaceutical assay, the proposed green RP-HPTLC method was found superior than the NP-HPTLC method for the determination of DLFX in pharmaceutical products.

## 4. Conclusions

A green RP-HPTLC method was developed and validated for the determination of DLFX in pure form, commercial tablets and in-house developed SLNs compared with a NP-HPTLC method. SLNs of DLFX was prepared using a “single emulsion and solvent evaporation method” and characterized for particle size, polydispersity, zeta potential and entrapment efficiency. Both the methods were validated for the determination of DLFX. However, the green RP-HPTLC method was found more accurate, precise, robust and sensitive for the determination of DLFX compared with a NP-HPTLC method. Both the methods were successfully applied for the analysis of DLFX in commercial tablets and SLNs. The green RP-HPTLC method reproduced the pharmaceutical assay results within the prescribed limit compared with the NP-HPTLC method. Overall, the green RP-HPTLC method was found superior to the NP-HPTLC method for the pharmaceutical analysis of DLFX in commercial tablets and in-house developed NPs. Therefore, the green RP-HPTLC method can be successfully utilized for the pharmaceutical assay of DLFX in a wide variety of commercial formulations containing DLFX as an active constituent.

## Figures and Tables

**Table 1:** The composition and characterization data of DLFX-loaded SA-CS-SLNs (*n* = 3).

Formulation Composition		Characterization Parameter	
DLFX (mg)	10	Particle size ± SD (nm)	368.0 ± 5.2
SA (mg)	400	Polydispersity index ± SD	0.2 ± 0.0
CS (mg)	10	Zeta potential ± SD (mV)	19.2 ± 1.4
Pluronic F-127 (mg)	50	Entrapment efficiency ± SD (%)	80.4 ± 3.1

**Table 2:** Linear regression data for the calibration curve of DLFX for green RP-HPTLC and NP-HPTLC methods (*n* = 6).

Parameters	Green RP-HPTLC	NP-HPTLC
Linearity range (ng/band)	25−1000	50−600
Regression equation	Y = 33.62x − 184.11	Y = 23.90x + 201.55
R^2^	0.9996	0.9995
Slope ± SD	33.62 ± 1.62	23.90 ± 0.86
Intercept ± SD	184.11 ± 4.52	201.55 ± 6.73
Standard error of slope	0.66	0.35
Standard error of intercept	1.84	2.74
95% confidence interval of slope	30.77−36.46	22.38−25.41
95% confidence interval of intercept	176.16−192.05	189.72−213.37
LOD ± SD (ng/band)	8.54 ± 0.22	17.31 ± 0.51
LOQ ± SD (ng/band)	25.62 ± 0.66	51.93 ± 1.53

**Table 3:** Accuracy data for green RP-HPTLC and NP-HPTLC methods (*n* = 6).

Conc. (ng/band)	Theoretical Content (ng)	Conc. Found (ng) ± SD	Recovery (%)	RSD (%)
Green RP-HPTLC method				
75	75	73.8 ± 1.4	98.4	1.89
300	300	296.9 ± 3.8	98.9	1.29
400	400	402.8 ± 7.0	100.7	1.74
500	500	505.1 ± 7.5	101.0	1.48
NP-HPTLC method				
150	150	144.2 ± 2.5	96.1	1.73
300	300	290.1 ± 4.8	96.7	1.66
400	400	383.7 ± 9.8	95.9	2.57
500	500	480.2 ± 12.7	96.0	2.65

**Table 4:** Precision data for the green RP-HPTLC and NP-HPTLC methods (*n* = 6).

Conc. (ng/band)	Repeatability (Intraday Precision)			Intermediate Precision (Interday)		
	Area ± SD	Standard Error	RSD (%)	Area ± SD	Standard Error	RSD (%)
Green RP-HPTLC method						
75	2316.7 ± 29.2	11.9	1.26	2442.2 ± 32.4	13.2	1.32
300	9776.1 ± 112.2	45.8	1.14	9598.2 ± 121.8	49.7	1.26
400	13,676.3 ± 219.3	89.5	1.60	13,754.6 ± 234.9	95.9	1.70
NP-HPTLC method						
150	3961.6 ± 69.7	28.4	1.75	4088.4 ± 75.3	30.7	1.84
300	7345.1 ± 126.7	51.7	1.72	7415.3 ± 137.2	56.0	1.85
400	9826.7 ± 241.6	98.6	2.45	9754.6 ± 255.5	104.3	2.61

**Table 5:** Robustness data for green the RP-HPTLC and NP-HPTLC methods (*n* = 6).

Conc. (ng/band)	Mobile Phase Composition (ethanol:water:ammonia Solution)			Results		
	Original	Used		Area ± SD	% RSD	R_f_
Green RP-HPTLC method						
		5.2:3.8:2	+0.2	9614.8 ± 106.9	1.11	0.83
300	5:4:2	5:4:2	0.0	9589.8 ± 104.1	1.08	0.84
		4.8:4.2:2	−0.2	9454.3 ± 114.8	1.21	0.85
NP-HPTLC method						
Mobile phase composition (ethyl acetate: methanol: ammonia solution)						
		5.2:3.8: 2	+0.2	7374.6 ± 122.7	1.66	0.43
300	5:4:2	5:4:2	0.0	7298.4 ± 116.5	1.59	0.44
		4.8:4.2:2	−0.2	7153.7 ± 134.4	1.87	0.45

**Table 6:** Assay of DLFX in marketed tablets and SLNs formulation by the green RP-HPTLC and NP-HPTLC methods (*n* =3).

Samples	Theoretical Content (mg)	Content Found (ng) ± SD	Assay (%)
	Green RP-HPTLC method		
Marketed tablets	50	49.1 ± 1.0	98.2
SLNs	50	50.5 ± 1.1	101.0
	NP-HPTLC method		
Marketed tablets	50	47.2 ± 1.2	94.4
SLNs	50	47.5 ± 1.3	95.0
